# Anticipating and suspending: the chronopolitics of cryopreservation

**DOI:** 10.1057/s41292-024-00332-3

**Published:** 2024-07-11

**Authors:** Thomas Lemke

**Affiliations:** Institute of Sociology, Goethe University Frankfurt am Main, Theodor-W.-Adorno-Platz 6, 60323 Frankfurt am Main, Germany

**Keywords:** Cryopreservation, Cryopolitics, Suspended Life, Latency, Potentiality, Anticipation

## Abstract

The article brings together two disparate and so far largely disconnected bodies of research: the critical analysis of cryopreservation technologies and the debate on modes of anticipation. It starts with a short review of the state of the research on the concept of cryopolitics. In the next part I will suggest two revisions. I will problematize the idea of latent life and the focus on potentialities that have been central to the research on cryopolitics so far, proposing to shift the analytic frame to suspended life on the one hand and to modes of anticipation on the other. I argue that cryopreservation practices are part of contemporary technologies of anticipation. They are linked to a politics of suspension by mobilizing a liminal biological state in which frozen organisms or biological material are neither fully alive nor ultimately dead. This seeks to avert and/or enable distinctive futures by extending temporal horizons and keeping vital processes in limbo.

The final power would thus be less one of imagination than of anticipation, so much so that to govern would be *no more than* to foresee, simulate, memorize the simulations; […] (Virilio 2006, p. 157, emphasis in orig.).

In the past ten years, a number of STS scholars have proposed the term “cryopolitics” or “cryopower” to capture the profound socio-material changes introduced by technologies producing low temperatures (Friedrich and Höhne 2014; Kowal and Radin 2015; Radin 2017; Friedrich 2017).^1^ The notion seeks to address the radical and ongoing transformation of temporal trajectories and spatial configurations in contemporary societies engendered by cryopreservation practices. In this understanding, cryobiological processes fundamentally affect the politics of life in the twenty-first century. They undermine conventional understandings of life and give rise to novel modes of controlling, enhancing and processing organic matter.

This article seeks to explore and further advance the “cryopolitical account” (Peres 2019, p. 77) by connecting it to the debate on modes of anticipation. This growing literature has emerged in the past two decades in STS and beyond, arguing for the need to explore how different futures are enacted through socio-material assemblages (Adams et al. 2009; Anderson 2010; Alvial-Palavicino 2015; Poli 2017; Davis and Groves 2019). Bringing together these so far largely disconnected bodies of research, I propose the concept of a politics of suspension. It builds on the idea of cryopolitics but shifts the analytic focus to the chronopolitical strategies enacted by cryopreservation and cryobanking practices that modify and mould the order of time to accommodate future events.

I start with a short review of the state of the debate on cryopolitics. The next part suggests two revisions. It problematizes the idea of “latent life” as well as the focus on potentialities that have been central to the literature so far, proposing to shift the analytic frame to “suspended life” on the one hand and to modes of anticipation on the other. I argue that cryopreservation practices are linked to a politics of suspension by mobilizing a liminal biological state in which frozen organisms or biological material are neither fully alive nor ultimately dead. They are an integral part of contemporary technologies of anticipation as they seek to avert and/or enable distinctive futures by extending temporal horizons and keeping vital processes in limbo.

## Reassessing cryopolitics: a brief review of the debate

The term cryopolitics was first introduced almost twenty years ago by Michael Bravo and Gareth Rees to draw attention to the increasing geostrategic importance of the Arctic region as melting polar sea ice opens up new political conflicts over material resources (Bravo and Rees 2006; Haverluk 2007; Bravo 2017). In the past decade, however, scholars have proposed a more comprehensive understanding of the term that directly follows Foucault’s analytics of biopolitics. Friedrich and Höhne (2014) and Kowal and Radin (2015; Radin and Kowal 2017) have argued that cryopolitics is an extension of the Foucauldian concept. Foucault famously contrasts biopower with sovereign power. While the latter is characterized by taking life or letting live, the former operates by technologies that foster life or let die (Foucault 2003, p. 241). Cryopolitics marks an important intensification of the biopolitical problem space as it is organized around the imperative to “make live and *not* let die” (Friedrich and Höhne 2014, p. 2, emphasis in orig.; Kowal and Radin 2015; Friedrich 2017; Radin and Kowal 2017).^2^ Thus, cryopolitics is defined by interrupting processes of development and decay, opening up a “unique biological state between life and death” (Neuman 2006, p. 260).

In this understanding, cryopolitics serves as a “theoretical frame brought into existence by the practice of freezing” (Kowal and Radin 2015, p. 68). It does not work as a conceptual alternative to the classical understanding of biopolitics but rather represents “a mode of Foucault’s biopolitics” (8ibid.; Friedrich and Höhne 2016; Radin and Kowal 2017). While this reading stresses continuity and consistency, the concept of cryopolitics also significantly enlarges the original understanding of biopolitics. In line with empirical insights and theoretical propositions by STS scholars and many other researchers investigating the impact of contemporary biosciences, the analytics of cryopolitics is marked by three important extensions. First, it shifts the focus of investigation beyond the disciplining of the individual body and the regulation of the population—the “two poles of development” (Foucault 1978, p. 139; 2003) in Foucault’s understanding of biopolitics. Cryopolitics opens up the analytic frame to include the control and enhancement of biological matter. Beyond the individual body of a human subject and the collective body of the population, it includes “bits and pieces from human bodies” (Hoeyer 2017, p. 207) such as gametes, tissue or DNA. Secondly, the concept of cryopolitics undermines any attempt to restrict biopolitics to “the vital characteristics of human existence” (Rabinow and Rose 2006, pp. 197–98; Rose 2007). Rather, it attends to the “totality of life” (Friedrich and Höhne 2014, p. 38; Friedrich 2020a, p. 247) and the multiple ways in which biopolitical mechanisms also affect nonhuman species, seeking to govern animal and plant life (Haraway 2008; Friese 2013; Wolfe 2013; see also Lemke 2021). Third, while the colonial legacy of biopolitics only plays a minor role in Foucault’s work, the analytics of cryopolitics often engages with colonial and racialized rationalities underpinning cryopreservation practices. One important area of research has been the International Biological Program (IBP) that ran from the 1960s through to the mid-1970s. Using mechanical laboratory freezers and techniques of cold storage, the anthropologists, biologists and physicians involved in the program collected hundreds of thousands of blood samples from indigenous communities in many countries, whose peoples were considered to be both unchanged by civilization processes and threatened by extinction. These collections were assembled in order to determine biological traits of individuals and populations often conceived of as ‘primitive’ to promote knowledge for the future of mankind. However, as TallBear notes, this research initiative (as well as many others with similar objectives) that claims to preserve indigenous DNA for the study of human diversity “is predicated on indigenous death” (TallBear 2017, p. 182; Radin 2013; Kowal et al. 2013; Kowal and Radin 2015; Radin 2017).

In the past decade, the concept of cryopolitics has attracted a lot of academic interest, especially after the publication of an edited volume on the subject by Radin and Kowal (2017). *Cryopolitics: Frozen Life in a Melting World* contained a number of important contributions covering topics such as biosecurity (Keck 2017), global food chains (Friedrich 2017) and species conservation (van Dooren 2017; Chrulew 2017; Kirksey 2017). In the wake of this seminal publication, the “cryopolitical analytic” (Radin and Kowal 2017, p. 4) was fruitfully extended to other relevant research fields. It has been used to analyse the control of temperature in urban spaces (Höhne 2018), to investigate the governance of frozen seeds repositories (Peres 2019), to assess the impact of cryogenic technologies in reproductive policies in Scandinavian countries (Kroløkke et al. 2019), to study the practices of egg cell freezing and the potential use of cryopreserved oocytes for biomedical research (Friedrich 2020b), to follow resurrection projects in Russia that seek to bring the mammoth back to life (Wrigley 2021), and to trace the technoscientific networks of human milk donation and banking in Spain (Romero-Bachiller and Santoro 2023).^3^

The “cryopolitical framework” (Kroløkke 2019, p. 541; Peres 2019, p. 84) often puts forward two fundamental and interconnected claims. Firstly, cryopolitics is characterized by “the perpetual deferral of death” (Kowal and Radin 2015, p. 68; see also Radin and Kowal 2017, p. 7) and draws on a state of “latent life” (Friedrich and Höhne 2014; Kowal and Radin 2015; Radin 2017; Kroløkke 2019; Romero-Bachiller and Santoro 2023). According to this reading cryopreservation practices make it possible to store organic material by cooling it to sub-zero temperatures for an indefinite period of time, resulting in a “life *without* death” (Kowal and Radin 2015, p. 69, emphasis in orig.). Secondly, the concept of cryopolitics designates the “potential of life or life forms that had been redirected in time through the use of low temperature” (Radin 2017, p. 4; 2013; see also Friedrich 2020b, p. 340). In this understanding, cryopreservation practices store potentialities available for future use, opening up scientific or medical perspectives as well as commercial options.

In the following, I propose a two-fold analytical shift to clarify and complement the original reading of cryopolitics. First of all, I will argue that in order to conceive the mode of operation of cryopreservation practices, “suspension” is more appropriate than “latency”. In contrast to “latent life”, “suspended life” accounts for the deferral of both death and life and better captures the liminal biological state of frozen organic material. Building on and extending the existing debate on cryopolitics, I seek to offer a conceptual clarification that suggests shifting the analytic focus from latency to “suspended life” (Lemke 2022). The idea of suspension in cryopreservation practices has been fruitfully explored before in conceptual reflections (Hoeyer 2017) as well as in empirical studies (Romero-Bachiller and Santoro 2023), but so far it has not been consistently distinguished from the notion of latency and its chronopolitical dimensions still lack a systematic consideration. Secondly, I suggest situating cryopreservation practices within the current “regime of anticipation” (Adams et al. 2009; Mackenzie 2013; see also Dolez et al. 2019). Analysing how cryotechnologies are mobilized within anticipatory rationalities displaces the promissory focus on potential with a practical interest in addressing future concerns and catastrophic risks.

## The limits of latency

Radin has emphazised the usefulness of the term “latent life” to denote the ability of cryopreserved cells to halt metabolic processes, putting living entities into a state of stasis—opening up the prospect of returning them to 'normal' development in the future. Cryopreserved biomaterial unsettles the idea of life as a linear and continuous process, disturbing the conviction that the cessation of vital processes inevitably leads to death. According to Radin, the conceptual focus on latency is particularly fruitful to “describe a huge range of concealed forces” (2017, p. 4), articulating Freud’s analysis of the psychic apparatus as well as Marx’s understanding of “latent capital” and Said’s critique of “latent orientalism” (ibid.). Moreover, the reference to latency emphasizes the fictive and speculative dimension of cryopreservation, as cryobanks not only assemble and preserve organic material for contemporary usages but also mobilize and explore purposes “as yet unknown” (Radin 2017, p. 55).

However, the focus on latency comes at a price. While the mobilization of the concept sensitizes to the deferral of death and the vital potentialities attached to cryopreserved material as well as their promissory nature, it also invites conceptual misunderstandings and involves analytic risks. The notion of latent life might nurture the idea of a “cryoexceptionalism”^4^ that focuses exclusively on (artificial) cold without taking into account other forms of ametabolic life and similar modes of biotechnological intervention. Cryopreservation practices are not unique in deferring death or in moulding temporal pathways. Apart from coldness there are several other (adverse) natural conditions that may engender this “peculiar state of biological organization” (Clegg 2001, p. 613): desiccation, lack of oxygen and high salt concentration (or a combination of them) might also result in a state of a “*temporary death*” (Neuman 2006, p. 260, emphases in orig.; Keilin 1959, p. 167; Crowe 1975; Clegg 2001; see also Lemke 2023a).

Also, it has to be noted that practices of cryopreservation represent an integral element of the technoscientific arsenal rather than possessing an outstanding quality. Like many other biotechnological interventions, they replace natural developmental processes by “artificial synchronization” (Landecker 2010, p. 220). Hannah Landecker has shown that biotechnologies are characterized by the power to stop and restart vital activities, to mobilize and manipulate cells, and to isolate and separate tissue culture so that it can 'live' for an indefinite span of time (Landecker 2005, 2007). As a result, biological matter is conceived of as plastic and flexible instead of stable and unchanging. Thus, “biotechnology changes what it is to be biological” (Landecker 2007, pp. 223–224). The “new biology” relies on the rearrangement of parts instead of focusing on whole organisms. It operates by arresting differentiation and decay and is based on a plasticity that curbs and tames capacities, represses spontaneous development and regulates vital dynamics.^5^ Thus, instead of insisting on the exceptional nature of cryopreservation practices, a more situated account is needed that locates them within the larger field of the new biology without denying their specifics and their practical and infrastructural relevance to many other biotechnological fields.^6^

The concept of latent life put forward in the literature on cryopolitics not only risks encouraging an analytic tendency towards cryoexceptionalism, but also suffers from conceptual problems. As I have argued elsewhere (Lemke 2022), the term nurtures a structuralist opposition of manifest and latent life and conceives of life as an inherent capacity of individual organisms.

The diagnosis of latency assumes the (hidden) existence of some residual life. Thus, the concept of latent life is characterized by a logical paradox, as the viability in ametabolic organisms or their body parts could be “recognized in retrospect only after they revive” (Roosth 2014, p. 64).

Unlike latent life, suspended life has been used much less frequently in the history of biology to describe the reversible state of metabolic stasis (Tirard 2010). These two terms differ in how they conceive of cryogenic stillstand. While latent life focusses on a present but hidden capacity, suspended life addresses the liminality of cryopreserved organic material, articulating the curious ontological state of the in-between or neither-nor. In this understanding, it is not the deferral of death alone but rather the joint suspension of life *and* death that characterizes cryobiological interventions.

Suspended life captures the peculiarities of cryopreserved organic material better than latent life, for a number of reasons. First, resisting cryoexceptionalism it situates “interrupted life” (Anderson 2015, p. 379) induced by natural or artificial cold on a spectrum of other natural environments and alternative biotechnological options. Second, the notion of suspended life does not support the ontological idea of a residual life or a structuralist dualism between potential and actual life, but rather articulates the liminal quality of the processes at stake. While latency focuses on the biological properties of individual organisms, suspension deals with the network of ecological relationships with other organisms and the material environment that enable living things to exist and sustain their existence (Margulis 1998; Gilbert et al. 2012; Nicholson and Dupré 2018). Third, suspended life better attends to the normative underpinnings of cryopreservation practices. While it is certainly important to stress the possibilities and options they engender, it is essential to analyse the selectivities and inequalities they inscribe. I will come back to this aspect later in this article, but want first to turn to the way potentiality has been highlighted in the literature on cryopolitics.

## From potentiality to anticipation

The literature on cryopolitics has convincingly stressed the future-oriented, speculative and promissory dimension of cryopreservation. As Klaus Hoeyer notes, “cold objects have become understood as ‘laden with potential’ […] and this process opens new avenues for commercialization” (Hoeyer 2017, p. 206).^7^ In a similar vein, Charlotte Kroløkke and Anna Sofie Bach emphasize the “multiple potentialities” (2020, p. 294) attributed to cryopreserved ovarian tissue to manage fertility-related risks and to restore cultural understandings of normality and naturalness. To capture the latent capacities of cryopreserved material, Friedrich in his analysis of egg freezing practices proposes the term “option value”. In this understanding cryopreserved material “can be regarded as cold based option values that save possibilities of life for potential future uses” (2020b, p. 339). While options, possibilities and potentialities are certainly an essential element of cryopreservation, it seems necessary to rearticulate this analytical focus on “pure potential” (Radin 2015, p. 361) or a “horizon of expectation” (Radin 2015, p. 371) within a broader examination of how cryopractices avert and/or enable future trajectories.

In his contribution to the edited volume on cryopolitics, Frédéric Keck (2017) has noted that the stockpiling of antibiotics, vaccines and antivirals by means of artificial cold is critical to mitigate the effects of a pandemic outbreak or a bioterrorist attack. Drawing on the work of Andrew Lakoff and his joint publications with Stephen Collier on rationalities of preparedness, he situates these frozen repertories within strategies of anticipating possibly catastrophic future events (2017, pp. 131–133). While Keck only implicitly links the logic of anticipation to cryopreservation practices, this theoretical connection promises to provide an important extension of the cryopolitical perspective. Collier and Lakoff’s work on preparedness as a distinctive anticipatory rationality helps to illuminate key aspects of cryotechnological practices and their relation to a Foucauldian understanding of biopolitics.

Collier and Lakoff consider the emergence of what they call “vital systems security” as a “significant mutation in biopolitical modernity” (2015, p. 21, 2021). While classical biopolitics is concerned with the social body of a population and mechanisms to improve its well-being and prosperity, vital systems security targets the technologies and instruments of classical biopolitics that increasingly came to be problematized as sources of vulnerability and risk, setting the stage for a “reflexive biopolitics” (ibid.). Like classical biopolitics it seeks to foster the welfare and health of populations, but it does so by addressing a new object: material infrastructures, functions and services considered to be essential for maintaining collective life. Vital systems security goes beyond traditional forms of population security as it strives to prepare for and govern future emergencies of various kinds such as natural disasters, terrorist attacks, pandemic disease outbreaks and disruptions of critical infrastructures:

[T]hese two forms of biopolitical security differ in their objects of concern, knowledge practices, and norms. Whereas population security addresses regularly occurring events that are distributed over the population in predictable ways, vital systems security deals with events whose probability cannot be precisely calculated, but whose consequences are potentially catastrophic. Vital systems security does not rely on statistical analysis of past events to generate knowledge about security threats, but rather on the simulation or enactment of potential future events. Its interventions seek to increase the resilience of critical systems and to bolster preparedness for future emergencies. (Collier and Lakoff 2015, p. 22; see also Folkers 2018, pp. 214–21; 343–352; Collier and Lakoff 2021, p. 12)

Collier and Lakoff define “preparedness” as a political technology that “responds to the governmental problem of planning for unpredictable but potentially catastrophic events” (Lakoff and Collier 2010, p. 244). It provides a set of operational responses reducing vulnerabilities to ensure that vital systems continue to operate through and after the disastrous event. Many “frozen archives” (Anderson 2015) inhabit this logic of preparedness. Facing biodiversity loss, some of them include endangered and extinct animal and plant biospecimen. One prominent example of safeguarding plant material is the Svalbard Global Seed Vault (SGSV), which serves as backup for a large number of research institutes and agricultural gene banks around the world (von Verschuer 2021; Wolff 2021). Other cryobanks are devoted to the biological conservation of animal species (Kirksey 2017; Chrulew 2017) or to resurrection initiatives (Searle 2020; Wrigley 2021).

However, contemporary cryopreservation practices are limited neither to rationalities of preparedness nor to modes of vital systems security. Rather, they mobilize a set of diverse anticipatory technologies characteristic of “life in contemporary liberal democracies” (Anderson 2010, p. 792; Granjou et al. 2017). Derived from the Latin *anticipare* (literally: taking care of something ahead of time), anticipation defines a mode of future-making that authorizes actions in the here and now in the name of the future. It draws on a mode of reasoning that justifies measures and interventions in the present without laying claim to having proof that they will effectively avert the threats posed. In this understanding, distinctive rationalities or “logics of anticipation” (Anderson 2010) can be highlighted: in contrast to preparedness, precaution sets in “once a determinate threat has been identified”, meaning it acts “before the identified threat reaches a point of irreversibility” since “[t]he presumption is that delay may be far more costly to that life, even if absolute proof of impacts and effects is lacking” (Anderson 2010, pp. 788–789; see also Ewald and Utz 2002). The principle of precaution guides practices of cord blood banking in regenerative medicine (Liburkina 2023) and egg freezing for reproductive purposes (Lafuente-Funes 2023). Here, the issue is not a collective catastrophic event but rather some kind of individual insurance policy that seeks to address health-related or fertility-related problems. Lucy van de Wiel notes an important “shift from reproduction to fertility in IVF, in which treatment is not aimed at having a child at present but rather at the proactive management of a more speculative fertility throughout the life course” (van de Wiel 2020b, p. 306, also 322). In this light, egg freezing no longer responds to the reactive need for treating an already existing form of infertility but anticipates a future infertility (van de Wiel 2020b, p. 316).

Anticipation refers to particular futures that may not be known for certain but nonetheless must be acted upon. Technologies of anticipation structure the way we address contemporary challenges, problems and catastrophes by folding the future into the present.^8^ They might draw on artificial as well as natural forms of cryopreservation. Ice core research, for example, is one of the most important branches of paleoclimatology, an essential element of contemporary climate science. It reconstructs the Earth's climate history using data from natural sources such as tree rings, ice cores and ocean sediments that can be up to millions of years old. Martin Skrydstrup reported a few years ago on the recovery of an “almost two-mile ice core from the surface to the bedrock of the Greenland ice sheet” (Skrydstrup 2017, p. 73), noting that this successful project made it possible to go back 128,500 years. The drilling of the ice core provided evidence of climate periods that were up to eight degrees Celsius warmer than today, allowing scientists to map the future of “global warming on the empirical terrain of ice” (Skrydstrup 2017, p. 72). Skrydstrup calls this form of reasoning “‘analogue anticipation’” as it “deploys past climatic epochs as analogues for the futures to come” (Skrydstrup 2017, p. 73). This anticipatory logic is based on observable natural data and measurable climate variables such as temperature, greenhouse gas or sea level. It complements contemporary modes of envisioning environmental futures that foretell the future by virtual modelling, focusing on probabilities, the compilation of statistical data and algorithmic prediction.

However, the most important cryo-related anticipatory logics today are neither analogue reasonings nor addressing (human-made) natures in the past, rather, they engage with the contemporary production of artificial cold. While the former anticipatory logic “predicts the past in order to envision the future” (Skrydstrup 2017, p. 70), the latter acts in the present to adapt to the future. This non-analogous anticipatory reasoning is not restricted to knowledge production but rather takes practical steps to avert the danger in question. Beyond identifying and imagining uncertain future trajectories, it aims at preparing for or precautioning what is yet to come.

## Cryopolitics as chronopolitics: dimensions of a politics of suspension

Recently, Friedrich has proposed an important “modification” (Friedrich 2020b, p. 340) to the original idea of cryopolitics. Rather than conceiving of cryopolitics as a mode of Foucauldian biopolitics, he suggests that it establishes a “new form or level of biopower” that differs significantly from the one “Foucault primarily had in mind” (2020a, p. 247). Instead of seeing it as operating on existing populations with concrete political objectives (e.g. reduction of diseases, rise of average life expectancy etc.), he now defines cryopolitics in terms of strategies to “safeguard *future* populations and their *possible* purposes”, converting the classical cryopolitical imperative into the new proposition: “to be *able* to make life and *not* let die” (2020a, p. 247, emphases in orig.; 2020b, p. 340).

While this revision makes it possible to shift cryopolitical inquiries to practices of future-making by means of artificial cold, it remains unclear how exactly cryopreservation practices relate to the future. To address this challenge, it is essential to displace the figure of the (human) population in the analysis of cryopolitical strategies by taking into account vital systems, critical infrastructures and more-than-human ecologies. Cryopreservation practices share with other technologies of anticipation the imperative to act in the present in order to accommodate the future, but also differ in important aspects from most strategies of preparedness or modes of precaution. As Leon Wolff (2021) has noted, cryopreservation practices do not just take action ahead of time but also work directly on temporal horizons by maintaining options and keeping events reversible. Unlike, for example, predictive policing (Meijer and Wessels 2019) or material stockpiling (Folkers 2019), which anticipate future events by taking concrete measures in the present, they seek to prolong the present in the light of the future. Thus, cryopreservation practices enact a form of “chronopolitics” which seeks to govern “the difference between the future and the present” (Kaiser 2015, p. 166).^9^ They not only “buy time” (Hoeyer 2017, p. 207) but also rearrange temporal pathways and developmental processes.

Thus, cryopolitics might be better conceived of as a politics of suspension. It is less defined by the potentialities of the cold but rather characterized by the (re-)ordering of temporal structures and social rhythms. This “temporal politics” (Opitz and Tellmann 2015) governs how past, present and future relate to each other, focusing on “the making of order by the marking of time” (ibid.: 108). The politics of suspension does not react to an uncertain future, seeking to adapt to it, but rather acts directly on temporal horizons by extending the present. It operates by “the principle of ‘whenever’” (Lemke 2023b), keeping events in limbo. It aims to avoid irreversible pathways and trajectories by prolonging the present—to determine when it ends, when it becomes a matter of the past.^10^

The politics of suspension is part of a larger shift in (bio)political rationalities. This transformation has at least two important aspects. The first refers to the emergence of a “reflexive biopolitics” (Collier and Lakoff 2015, p. 21) or to “ontopower” in Brian Massumi’s terms (Massumi 2015). Massumi diagnoses a major shift that is displacing technologies of power, redirecting them from a biopolitical horizon to what he calls “ontopower”. While biopower was based on the figure of the population that could be governed by calculative planning, demographic data and standardised forms of intervention, contemporary societies are faced with “a nonstandard environment, characterized by an ever-presence of indiscriminate threat, riddled with the anywhere–anytime potential for the proliferation of the abnormal […] in a world that is in a permanently farfrom-equilibrium critical condition” (Massumi 2015, p. 26).

The second aspect corresponds to a certain rupture of the modern narrative replacing the ideas of progress and an open future by the “sens of a stalled present” (Scott 2014, p. 6): “Time, in short, has become less yielding, less promising than we have grown to expect it should be. And what we are left with are *aftermaths* in which the present seems stricken with immobility and pain and ruin; a certain experience of temporal *afterness* prevails in which the trace of futures past hangs like the remnant of a voile curtain over what feels uncannily like an endlessly extending present” (Ibid.: 6, emphases in orig., see also Staab 2022, pp. 72–106).

Within this new constellation the mobilization of suspended life allows to extend the present. The establishment of cryobanks for various purposes might make it possible to keep options open by expanding the duration of the present, but it often also contributes to delaying necessary decision-making or keeping problems in limbo instead of tackling them. Enacting a logic of anticipation, cryobanks seek to mitigate the destructive and disruptive consequences of (future) catastrophes instead of stopping the course of events that leads to them. One example is the increasing importance of cryopreservation for safeguarding biodiversity. More and more zoos, museums, and other conservation bodies are investing in infrastructures to cryogenically preserve tissue or DNA from endangered or already extinct animals. However, these efforts to counteract the sixth mass extinction are having ambivalent effects. In their ethnographic study of the Frozen Ark Project in the United Kingdom, Breithoff and Harrison (2020) observe an important shift in the way the project operates. Whereas Frozen Ark was once conceived as a static and passive repository for an uncertain future, the scientists involved now affirm a more active role for the collection, recognizing the value of genetic material for potential revitalization strategies and future de-extinction programs. The narrative of storing and safeguarding is increasingly being replaced by notions of speculation and investment, the concept of an ark taking a back seat to the imaginaries of a bank. As Breithoff and Harrison note: “The Frozen Ark counterintuitively depends upon the future biodiversity loss which it works against, but simultaneously anticipates, in its present operations” (2020, p. 50; Chrulew 2017).^11^

Moreover, the politics of suspension often endorses ‘conservative’ tendencies in gender relations. One example is the cryopreservation of oocytes, which addresses women as responsible for managing risk and planning reproductive futures. Instead of challenging workplace policies as well as the gendered division of labour to better align work and family relations, egg freezing enacts a technological fix by mobilizing the idea of an individual ‘biological clock’ that must be reconciled with professional life (Waldby 2015; van de Wiel 2015, 2020a), enforcing dominant and gendered concepts of motherhood and parenting (Myers 2017; Baldwin 2019).^12^ Similarly, a number of studies have provided evidence of how the history of cryonics is intimately connected to “visions of future male power” (von Verschuer 2021, p. 153) which assume that cryo-based and non-sexual modes of reproduction will prevail in the future, promoting “white American ideals and rhetorics of pioneering, frontierism and ahistoricism” (Farman 2020, p. 150).^13^

The politics of suspension privileges a liberal understanding of time as the object of responsible management and the cultivation of prudence and foresight. In this light, time appears to be “an instrumental medium at the disposal of the entrepreneurial subject” (Waldby 2019, p. 27). To focus on the politics of suspension makes it possible to map the uneven vulnerabilities cryopreservation practices enact. It inquires how suspended life is linked to forms of “slow violence” (Nixon 2011), investigating how the “economies of harm, suffering and insecurity within liberal societies” (Anderson et al. 2020, p. 629) generate different but constitutively related temporalities in which some are able to cultivate future-oriented anticipatory capacities while others are confined to a permanent present without any perspective of change and improvement.

Finally, it has to be noted that cryopreservation practices do not just add options or enact a surplus of potential, they potentialize and depotentialize at the same time—or rather, they potentialize by depotentializing: the taming and curbing of developmental pathways and vital processes in order to realise some objectives excludes or marginalizes alternative options. Thus, there is a hidden normative agenda inscribed in the politics of suspension that needs to be uncovered. Which issues become “matters of care” (Puig de la Bellacasa 2017) to be addressed in cryopreservation practices, and who has the power to define them? What needs to be stored in cryobanks and what may get lost or go extinct? For what futures is the frozen material of animals and plants preserved and what are the social and physical landscapes they will inhabit (Laboissière 2019, p. 67)? Who participates in the decision-making process? What social and ethical values materialize in these anticipatory technologies, and how do they protect, enhance or save some forms of life while endangering, marginalizing or destroying others?

It is important to attend to the selectivities the politics of suspension enacts, rendering some problems visible while obscuring others. There are three dimensions to this mode of (de-)valuating life. As we already saw, it needs to be specified what is worth preserving by artificial cold. What species need to be protected from the risks of extinction, what kind of tissue or cells are considered too precious to expose them to the ‘normal’ course of development and decay? Moreover, the politics of suspension privileges a specific form of conservation. Instead of engaging with embodied, situated and finite living entities in concrete ecosystems, it focuses on organic material and genetic information in cryobanks to “secure life against the political and environmental vagaries of living itself” (Chrulew 2017, p. 288). Finally, the government of suspended life raises the question of who controls the capacities to shape the future within the present, disposing over the “‘means of anticipation’” (Aykut et al. 2019, p. 4; see also Groves 2017). For example, it is mainly white well-educated women in the Global North with higher income who have frozen their oocytes to delay childbearing. Currently, the option to cryopreserve eggs increases rather than diminishes social inequalities as the majority of women and couples in the world do not have access to reproductive medicine at all (Inhorn 2017; Myers and Martin 2021, pp. 5–6).

## Conclusion

In this article, I have reviewed the current state of the debate on cryopolitics and proposed to shift the conceptual accent from the preoccupation with latency and potentiality to suspension and anticipation. I have then spelt out important dimensions of a politics of suspension based on the mobilization of cryopreserved biological matter to address future issues and concerns.

Understanding cryopreservation practices as “technologies of anticipation” offers a number of analytic advantages. Firstly, this conceptual proposition goes beyond the exclusive focus on the “urgency of disaster” (Collier and Lakoff 2021, p. 11) we see in many studies of anticipatory practices as it also includes the concerns and challenges of “daily life” (Granjou et al. 2017, p. 8) within the anticipatory horizon. Thus, conceptualizing cryopreservation practices as technologies of anticipation offers a more comprehensive picture that can address the exceptional and the mundane within a single analytical frame. This reading not only advances the debate on modes of anticipation but is also attentive to forms of suffering and harm intimately tied to the experience of everyday life, such as “crisis ordinariness” (Berlant 2011). Secondly, this conceptual move makes it possible to study the interplay between individual and collective subjectivities, not only concentrating on vital systems security or issues of political government but also including other and more decentralized forms of (self-)government used to determine and manage health and fertility risks for oneself and family members. Finally, situating cryopreservation practices within contemporary technologies of anticipation opens up the question of the conditions under which an issue becomes a “matter of concern” (Latour 2004) or an 'emergency' to be fixed by cryotechnologies. Aligning the analytics of cryopolitics to the debate on modes of anticipation points to the selective formats and uneven vulnerabilities cryopreservation practices enact and their normative underpinnings, which differentially valorize certain forms of life at the expense of others. In this sense, the politics of suspension still follows the old biopolitical rationality to make live and let die by claiming to make live.

## Footnotes

1 The word “cryos” derives from Ancient Greek (κρύος krýos) and means “ice” or “cold”.

2 All translations from German are mine. The formula (“to make live and not let die”) was probably first introduced by the philosopher Boris Groys to denote the biopolitical utopia of bodily immortality by technological means and social organization in early twentieth century Russia (Groys 2005, p. 8; see also Bernstein 2019).

3 Mostly independently of the work on cryopolitics, scholars in social theory and media studies in the past decade have suggested that we should attend to the cultural and social dimensions of temperatures and how they are implicated in distinctive strategies of power (Starosielski 2016, 2021; Beregow 2019a). These “critical temperature studies” (Starosielski 2021, p. 8) trace the material effects of thermal arrangements within and beyond freezing and cooling practices and study the entanglements of temporalities, temperature and matter. This “thermopolitics” (Beregow 2019b; Clark 2019) or “thermopower” (Starosielski 2021, p. 3) designates “the enactment of social and political power through thermal manipulation” (2021: 3). In this understanding, instead of being purely natural phenomena, heat and cold are technologically mediated and their modulation provides new forms of social, biological and environmental control.

4 I propose the notion of cryoexceptionalism by drawing on the debate on “genetic exceptionalism” that critically analyses how genetic information in medical research and clinical practice is regarded as privileged and treated “as different from other health-related information” (Murray 1997, p. 71), fueling misplaced determinist and reductionist accounts of genetics.

5 As Sarah Franklin notes in her empirical study on stem cell research: “It is very important that the stem cell colony has a systematic integrity, that it is clearly differentiated, and well characterized. This stability and uniformity is ‘good form and function’ for a cell line. But it must be singular and identical, and not developing new parts. It must be almost the opposite of the old developmental biology in that it should be both reproducing and staying the same—ironically so its ‘plasticity’ can be harnessed” (Franklin 2007, p. 66). Here, the immortality of the cell line rigorously depends on its 'non-development', on the reproduction of sameness, reframing life as a series of interruptions instead of a continuous sequence.

6 Friedrich distinguishes “cryofacts” from what Nicole Karafyllis has termed “biofacts” (Karafyllis 2003, 2007). While the latter refer to “man-made things that grow”, the former seek to prevent development and decay: “In contrast to biofacts, cryofacts are not ‘things that grow,’ but things that may grow; and for this possibility they are precisely *prevented* from growing” (Friedrich 2020b, p. 339, emphasis in orig.). However, “cryofacts” are nothing but the result of a form of biological control that is not specific to cryotechnologies but pertains to other forms of biotechnological intervention. Thus, the conceptual juxtaposition is not convincing, as cryofacts may be regarded as a particular form of biofacts whose metabolic processes are suspended.

7 On the meanings and dimensions of potentiality in the biosciences see Taussig et al. (2013).

8 The idea of a fold was prominently put forward by Deleuze (1992) in his interpretation of Leibniz’s philosophy and concept of the monad as an entanglement of space, movement, and time. Gramelsberger et al. (2021) propose the term for the analysis of cryotechnologies as a “time(re)configuration technique”.

9 The term chronopolitics was famously proposed by Paul Virilio (2006, 2012). Virilio argues that the politics of space (geopolitics) has been displaced by a politics of time (chronopolitics) as technologies of war and speed have fundamentally transformed social, economic and political relations in contemporary societies. My use of the term differs from Virilio’s understanding as chronopolitical strategies are not restricted to acceleration and speed but might also include deceleration and suspension. Also, I do not share Virilio’s foundational separation of time and space (for a more extensive critique of Virilio’s concept of chronopolitics see Klinke 2012; for an overview on his writings on security, technology and society see Lacy 2014). The term chronopolitics has been used before to characterize cryopreservation practices. According to Leon Wolff, it articulates “the chrono-political problem of establishing a specific temporal order in which possibilities are maintained and events can become reversible” (Wolff 2021, p. 80). To attend to the temporal dimensions and normative underpinnings of cryonics—the programmatic idea of freezing clinically dead bodies to resuscitate them in the future—, Abou Farman (2020) turns to Dana Luciano’s notion of “chronobiopolitics” (2007). Conjoining “the cryopolitical to the chronopolitical” (2020, p. 134), he seeks to analyse how cryonic operations renegotiate life and time.

10 In his study of cryonics, Farman also employs the term “politics of suspension” (2020, p. 123). However, in his analysis, the notion refers not to strategies for securing reversibility and a prolonged present, but to tensions and contradictions that arise from a “secular space in which the promise of the indeterminate future can take material embodiment in the figure of the cryopreserved patient” (Farman 2020, p. 159).

11 Radin has identified a seemingly paradoxical strategy of “planned hindsight” by scientists working in the field of systematic taxonomy and conservation biology in the 1980s. Their efforts to coordinate, regulate and maintain early collections of frozen biospecimen were informed by the rationale “to organize the present in a way that appeared to anticipate the needs of future scientists” (Radin 2015, p. 361).

12 For an overview of the literature on the social and gender-related implications of egg freezing see Myers and Martin 2021.

13 The critical understanding of cyropolitics as a form of chronopolitics presented here also resonates with queer feminist interrogations of time as coherent, linear and progressive, seeking to envision alternative temporalities of being and belonging. Putting forward the notion of “chrononormativity”, Freeman (2010) has designated this self-naturalising concept of time as crucial to the making and marking of bodies and subjectivities in capitalist heteronormative societies. The mastering of cultural norms and social expectations relies on the ‘right’ allocation of time and the successful synchronisation of bodies. Thus, chrononormativity turns “naked flesh […] into socially meaningful embodiment through temporal regulation” (Freeman 2010, p. 3; see also Halberstam 2005; McBean 2013).
